# Experiences of community-dwelling older adults with chronic low back pain in Hong Kong and Switzerland – A qualitative study

**DOI:** 10.3389/fresc.2022.920387

**Published:** 2022-08-17

**Authors:** Veronika Schoeb, Marceau Misteli, Crystal Kwan, Chris W. Y. Wong, Mandy M. P. Kan, Emmanuelle Opsommer, Arnold Y. L. Wong

**Affiliations:** ^1^ School of Health Sciences (HESAV), University of Applied Sciences and Arts Western Switzerland (HES-SO), Lausanne, Switzerland; ^2^ Department of Applied Social Sciences, The Hong Kong Polytechnic University, Hung Hom, Hong Kong SAR, China; ^3^ Department of Rehabilitation Sciences, The Hong Kong Polytechnic University, Hung Hom, Hong Kong SAR, China

**Keywords:** chronic low back pain, older adults, qualitative research, culture, healthcare services, community-dwelling

## Abstract

**Introduction:**

The prevalence of chronic low back pain (LBP) increases with age and older adults are more vulnerable to develop chronic LBP. A recent Swiss study has shown that 78% of community-dwelling older adults aged ≥65 years experienced chronic LBP. Similarly, a study in Hong Kong found that approximately 30% of people aged above 60 years experienced chronic LBP. The aim of this collaborative research project was to illuminate older adults' experiences of living with chronic LBP and its implication on older adults' daily life in Western and Eastern cultures.

**Methods:**

Twenty-five older people experiencing chronic LBP living in Switzerland or Hong Kong were recruited through health professionals or community centres. Using semi-structured interviews, participants shared their experiences regarding chronic LBP and its implications on their daily life. The interviews were recorded and transcribed “ad verbatim” in the original language. An inductive thematic analysis was used, using a qualitative data analysis software program (NVivo) and a shared code book in English. The Swiss and Hong Kong research teams engaged in collaborative analysis until a consensus was established, taking into consideration of cultural specificities. Ethical approval was obtained from the local ethic committees in both regions.

**Results:**

Themes were related to negative perceptions/experiences: (1) interferences of daily function; (2) pessimistic attitudes toward their conditions/prognosis; and (3) self-perceived burden related to families. Conversely, four themes revealed attributes to social roles: (1) maintaining their roles in families; (2) experiencing supports from family and friends; (3) being content despite LBP; and (4) enjoying social activities. Cultural differences between Switzerland and Hong Kong were related to social circles and offers from the healthcare system, influencing individual experiences and perceptions.

**Discussion:**

Although chronic LBP may negatively impact older adults, individual approaches as well as social and health system supports influence older adults' attitude toward their pain and self-management strategies. Developing effective and culturally sensitive interventions for an elderly population with chronic LBP can be challenging but essential for the development of innovative healthcare services tailored to the population's needs. The methodological approach used for this research project establishes the framework for developing and evaluating complex interventions.

## Introduction

The average human life expectancy of people aged 60 years or above is expected to increase continuously worldwide given the improved health care delivery and technologies (1). The United Nation has estimated that by 2050, of the multiple health issues, low back pain (LBP) will be the most prevailing health condition in older adults that leads to functional limitations and disability worldwide (2, 3). Several population-based studies (including Hong Kong) have estimated that the 1-year prevalence of LBP (regardless of chronicity) in community-dwelling older adults ranged from 13% to 50% (4, 5). Importantly, the prevalence of chronic LBP increases with age (6, 7) and older adults are more vulnerable to develop chronic LBP. Chronic LBP is defined as pain in or near the lumbosacral spine with or without leg pain that lasts for at least 3 months (8). A recent large-scale Swiss study has shown that up to 78% of community-dwelling older adults aged ≥65 years experienced chronic LBP (9), and that 17% of people from this age group have back pain several times a week, and another 17% several times a month (10). Similarly, a population-based study in Hong Kong found that approximately 30% of people aged above 60 years experienced chronic LBP (11). Older adults with chronic LBP experience problems in mobility, self-care, and emotions (9).

Multiple biopsychological factors have been attributed to the high prevalence of chronic LBP in older adults. Older adults are known to have high pain sensitivity because of age-related reduced brain activity responsible for endogenous (controlled by the central nervous system) pain modulation processing (12, 13), and lower pain thresholds (14, 15). The presence of comorbidities or spinal degeneration (e.g., lumbar spinal stenosis) in older adults may also worsen the physical function of patients with chronic LBP (16, 17), and reduce their treatment compliance (18). Compared to older adults without comorbidity, older adults with one or at least two comorbidities are 3 and 5 times more likely to have chronic LBP, respectively (14). Of various co-morbidities, psychological distress (e.g. depression and anxiety) is known to be an important risk factor for chronic LBP and LBP-related disability in community-dwelling older adults (19). Given the high prevalence of in older adults (20), and close association between depression and LBP, it is not uncommon to find older adults with chronic LBP and concomitant depression.

The high prevalence of comorbidities and frailty in older adults may result in very different perceptions and experiences of chronic LBP as compared to working-age adults (21). Older adults need to face multiple age-related physical and psychosocial challenges (e.g., deteriorated physical strength and mental capacity, comorbidity, the loss of loved ones, an altered social role, and financial constraints) that may compromise their LBP recovery and well-being (22). Additionally, chronic regional/widespread pain in older adults can increase their risk of psychological distress (23, 24) and social isolation, which in turn may compromise their health-related quality of life, and increases their risk of cardiovascular diseases and mortality (25). Furthermore, the chronic LBP experience in older adults may be modified by their way of living and social support (26). For instance, older adults residing alone may experience more pain and psychological challenges.

People living in Switzerland and Hong Kong have similar life expectancy: men's life expectancy at birth is 81.6 years in Switzerland and 82.1 years in Hong Kong, while women have a life expectancy of 85.4 years in Switzerland and 88.1 years in Hong Kong, making the top of the list of industrialised countries (27). While Switzerland has about a population size comparable to Hong Kong the two jurisdictions have different healthcare systems.

Switzerland's healthcare system is highly decentralised (28) as each of the 26 cantons has some power over key operations. The Swiss Federal Law on Compulsory Health Care (LAMal), however, requires all Swiss residents to buy private health insurance. The private health insurances are supervised by the state and the cantons, and provide coverage in case of sickness, accidents and maternity. Additional private insurance can be purchased to complement basic coverage (e.g. private hospitals, dental care) (28). While the quality of the Swiss healthcare system is high, it is one of the most expensive one in the world. As of the latest figures, Switzerland's health expenditure represented 11.3% as a fraction of gross domestic product (GDP) (29).

Hong Kong's healthcare system offers public hospital care comparable to the NHS (30), yet only requires 5.9% of Hong Kong's GDP (27). The Food and Health Bureau is responsible for resource allocation and healthcare policies, as well as supervising the Hospital Authority (HA) organised to serve the entire population with subsidised healthcare (31). A recent study investigating Hong Kong's longevity came to the conclusion that Hong Kong has the lowest cardiovascular mortality rate and few transport accidents due to low car ownership, low alcohol consumption and an excellent transport system (27). However, Hong Kong also has many high-quality private healthcare service providers in primary, secondary, and tertiary care settings to serve those who can pay for the medical expenses by themselves or paid by private health insurance.

Apart from the distinct healthcare systems, Western and Chinese cultures seem to deal differently with life challenges. In Western cultures, individualism is the social norm whereas “Chinese culture advocates collectivism” (32). Furthermore, Leung and Sung-Chan (32) argue that it is important for Chinese people to maintain a harmonious interpersonal relationship while simultaneously modify their attitude and behaviour to meet what is expected from them within their social system. This is in line with Leventhal et al.'s (33) perceptual-cognitive approach that explains how people vary in their representation of health and illness according to their socio-cultural background. According to Leventhal's theory, people with chronic pain make sense of their experience by drawing on their knowledge and beliefs (33). Representations and beliefs regarding illness and health are constructed within a social context, and therefore, it is interesting to know whether there are differences between older people with chronic LBP from Eastern and Western cultures.

Yet, as shown in this introduction living a long life does not necessarily mean living without chronic LBP. Given the complexity and adverse consequences of chronic LBP in older adults, it is essential for various stakeholders (e.g., allied health professionals or social workers) to gain an in-depth knowledge of the possible impacts of chronic LBP on older adults' daily life and overall experience of health. The findings can guide the development of more effective mono- or interprofessional pain management interventions for this largely heterogeneous group, as well as providing services adapted to the target population.

Against this background, the aim of this collaborative research project between Switzerland and Hong Kong was to illuminate older adults’ experiences of living with chronic LBP and its implication on their daily lives in Western and Eastern cultures.

## Methods

### Participants and data collection

Twenty-five older adults experiencing chronic LBP living in Switzerland or Hong Kong were recruited through health professionals (GPs, physiotherapists), or community centres. To be eligible for the study, participants were required to be aged 65 years or above, and have LBP in or near the lumbosacral spine with or without leg pain for at least 3 months in the last 12 months (8). Using semi-structured interviews, participants shared their lived experiences regarding chronic LBP and its implications on their daily life (34). The interviews were recorded in the local language (French or German in Switzerland, Cantonese in Hong Kong) and transcribed “ad verbatim” in their entirety in the original language (35). By using a qualitative approach, participants can share their experiences regarding chronic LBP, and evaluate its implications on their daily life. Pilot interviews were conducted to control suitability of the overarching questions. An interview guide (Appendix) was used to ensure transparency and validity of the approach (36). Interviews lasted for approximately one hour. In qualitative studies, the questions of sampling and sample size are recurrent. Using a purposive sampling strategy is considered appropriate for an exploratory qualitative study, as the intention is not to have a representative sample but to get an in-depth understanding of a specific phenomenon from a variety of people who can talk about it (37). Given the exploratory nature of this study, the number of participants was limited to maximum of 15 in each country. According to a recent empirical study (38), recurrency of themes with each additional interview (also known as code saturation) was reached after nine interviews. However, the authors recommended to use between 16 and 24 interviews to reach meaning saturation.

### Data analysis

An inductive thematic analysis was used using a shared coded book in English (39). The six steps described by Braun and Clarke (39) are as follows (p 87):

Step 1: Becoming familiar with the data (transcribing data, reading and rereading data, jotting down ideas),
Step 2: Generating initial codes (coding systematically across data set, collating data),
Step 3: Searching for themes (collating codes into themes, gathering data relevant to each theme),
Step 4: Reviewing themes (checking themes, generating thematic map),
Step 5: Defining themes (refining specifics of each theme and overall story of analysis, generate names of themes),
Step 6: Writing-up (selecting compelling extracts, final analysis, relating analysis back to research question and literature).

The particularity of this research team is that while English was the shared language, none of the interviews were conducted in English, but in French, German and Cantonese. Step 1 of the thematic analysis was done by each country team separately. Qualitative data analysis software program (NVivo, released in March 2020) was used for the coding and as mentioned above, the codebook was shared (Step 2). The Swiss and Hong Kong research teams discussed themes first separately (Step 3) before engaging in collaborative analysis until a consensus was established (Step 4), taking into consideration of cultural specificities. When defining themes (Step 5), country teams revisited the original data for confirmation. The write-up (Step 6) was done collaboratively, allowing for a final analytic step for theme construction. Thematic analysis across languages and cultural divides was an interesting endeavour and a methodological approach worth discussing about in an article later-on. Ethical approval was obtained from the respective local ethic committees in Switzerland (CER-VD No 2021-00241) and The Hong Kong Polytechnic University's Ethics Committee (No HSEARS20210128001).

Reflexivity was adopted throughout the study to ensure scientific rigor (36). Specifically, reflexivity is defined as a thoughtful and conscious process that includes continuous evaluation of subjective responses, interpersonal dynamics, and the research process itself (40). Additionally, a coding manual accessible to all members online was used throughout the data analysis to improve the transparency of the process and to help guide methodological steps.

## Results

Table 1 summarises the characteristics of all participants (*n* = 25). Seven men and six women in Switzerland, and 12 women from Hong Kong (aged 68 to 92 years) participated in the study. The majority of participants (64%; *n* = 16/25) was married, while six participants (24%) were widowed, and three divorced (*n* = 3; 12%). Two participants from Hong Kong were excluded because one participant had acute back pain and the other participant was only 62 years old. Participants in Switzerland were recruited through physiotherapy and medical private practices, while participants in Hong Kong were recruited in Community Centres where people gathered to attend classes and talks, and to meet friends. The socio-economic status in Hong Kong is rather low as evidenced by the fact that half of the Hong Kong participants live in public housing and receive support from the Comprehensive Social Security Assistance Scheme (CSSA). These data are not relevant in Switzerland as public housing does not exist and all adults older than 65 years receive a social security subsidy.

**Table 1:** Participants’ characteristics.

Region	ID	Gender	Age	Civil status	Previous professions	Current occupations/leisure activities
Switzerland	S01_M_68	m	68	Divorced	Mechanic, technologist	Playing guitar, walking/hiking group
	S02_M_92	m	92	Widower	Carpenter	Retired
	S03_M_75	m	75	Married	Factory, construction worker, gardener	Traveling, gardening
	S04_F_70	f	70	Married	Housewife, kiosk owner	Playing board games, walking
	S05_M_72	m	72	Divorced	Field service (sales)	Riding motorbike, walking, breeding spiders
	S06_M_73	m	73	Married	Police ambulance driver	Retired
	S07_M_73	m	73	Married	Law, trade	Working as a lawyer, store managing, gardening
	S08_F_75	f	75	Married	Law	Taking care of grandchildren, travel
	S09_F_85	f	85	Married	Farmer, cook	Retired
	S10_F_69	f	69	Divorced	Secretary	Volunteering
	S11_F_74	f	74	Married	Employee (university degree)	Working as a translator, housing, traveling, taking care of grand-children
	S12_F_74	f	74	Married	Employee	Volunteering, teaching, hiking, taking care of grand children
	S13_M_78	m	78	Married	Employee, law, judge	Volunteering, skiing, walking, gardening
Hong Kong	HK01_F_83	f	83	Widow	Housewife	Going to elderly centre, eating dim sum with friends, travelling
	HK02_F_68	f	68	Married	Housewife	Doing housework, doing exercise, texting with friends (WhatsApp), travelling with friends, going to elderly centre
	HK03_F_75	f	75	Married	Travel agent	Going to gym, doing exercise, fitness class, yoga class, climbing, swimming, walking, church activities, watching “Youtube”, texting with friends (WhatsApp)
	HK04_F_81	f	81	Married	Domestic helper	Doing exercise, going to elderly centre, eating dim sum with friends, visiting relatives, doing housework
	HK05_F_73	f	73	Widow	Clerk	Hiking, eating dim sum with friends, going to elderly centre, dancing, visiting elders, cycling machine
	HK06_F_75	f	75	Married	Factory worker	Walking, cycling, eating dim sum with friends, volunteer (visiting elders), taking grandchildren to school, going to elderly centre
	HK07_F_85	f	85	Widow	Housewife	Doing housework, walking, weaving/handicraft, eating dim sum with daughters, going to elderly centre
	HK08_F_80	f	80	Married	Tea seller/ factory worker	Volunteering (dancing class helper+ councillor's office + visiting elderly), doing exercise, eating dim sum, going to elderly centre, doing housework
	HK09_F_76	f	76	Married	Hardware business owner	Doing exercise, using smartphone, doing housework, browsing internet (Youtube), going to elderly centre, going to church and park
	HK10_F_72	f	72	Married	Cleaner	Doing exercise, doing housework, going to elderly centre, phonecall with friends, eating dim sum with children
	HK11_F_81	f	81	Widow	Grocery store worker	Doing housework, doing exercise, going to elderly centre and Po Leung Kuk, taking photos, walking in the park
	HK12_F_84	f	84	Widow	Accountant	Doing exercise in the park, reading books and newspaper, phonecall with friends

Analysing the participants' professional backgrounds in the two regions, nearly half of the Swiss sample were white-collar workers (clerical, professional and managerial jobs) before retirement, whereas most participants in Hong Kong were blue-collar workers (cleaner, factory worker, hardware and grocery store workers) before retirement. Hiking, gardening, traveling and volunteering were the most common current leisure activities of Swiss participants, while doing exercises, walking/hiking and eating dim sum were the most frequent activities done by Hong Kong participants. Eating dim sum (going for a tea in an European setting) is an important social activity in Hong Kong. As the current study adopted convenience sampling, it happened that only female participants were recruited in Hong Kong while 50% of Swiss participants were men.

Despite different socioeconomic and cultural context, our analysis revealed some common themes but as hypothesized also some important differences.

The common themes were related to negative perceptions/experiences: (1) interferences of daily function (including sleep); (2) pessimistic attitudes toward their conditions/prognosis; and (3) self-perceived burden related to families and avoidance of talking about their pain with families.

Conversely, four themes revealed attributes to social roles: (1) maintaining their roles in families (e.g., housework); (2) experiencing supports from family and friends; (3) being content despite LBP; and (4) enjoying social activities. Cultural differences between Switzerland and Hong Kong were related to social support networks and opportunities/offers from the health and social system, influencing individual experiences.

### Negative experiences

#### Interferences of daily function

Chronic LBP affected older adults' activities in daily life, since many of them reported difficulties in walking, sitting and prolonged standing. Some older adults had trouble in doing housework and/or carrying heavy objects, while others did not go out or drive any more.

Sometimes I feel exhausted when I am doing housework, for example, when I stand for a long time. I need to do it for a while and sit for a while. (HK04_F_81)

I can do this whole part of the house for you, including the dusting, all that, clean. But I can do it in half a day, I would say, because I'm a little bit… If you see something that's dirty, you go all the way, you finish your job. But now I can't. I do half, half a day, and the next day, another half day. (S12_F_74)

Well, now, over time, for example, I told you I was singing in a big chorus. So, the fact of standing for a long time and carrying the score. And then to be carried away by the emotion, the music and everything that goes with it, it's starting to become difficult for me, but I haven't decided yet that I want to stop because of that, but sometimes I say to myself I'd rather have a lectern to put my score on it. (S13_M_78)

I used to take the car, to have a drink at noon or 11am. Now I don't go out anymore. I hardly drive either. My wife and daughter tell me “You have to give your driver”s licence back, because you don't drive any more”, I say well, we'll see, it's still up to me, for the moment. (S06_M_73)

Activities of daily living were not possible for participants in the same way as before or needed to be adjusted to the current possibilities and capacities. All participants realised that there were adaptations to be made, either to interrupt activities (HK04_F_81) as they were not able to do them without taking a rest, use more time to accomplish certain activities (S12_F_74) or use assistive devices to help overcome limitations (S13_M_78).

#### Pessimistic attitudes toward their conditions/prognosis

Participants expressed that there was no cure for them, that the aging process would not allow them to be free of pain and that pain would actually stay with them “till the end of her life”. It became also evident in our data, that worries were closely linked to back pain, independent of the participants' origin and cultural context.

I am worried, because many people said it can't be cured. It's hard to cure it at my age. (HK10_F_72)

I can do nothing. I feel … sometimes desperate because of the incurable condition. (HK03_F_73)

I have always back pain, so that's what I am ending up being worried about. (S06_M_73)

#### Self-perceived burden related to families and avoidance of talking about their pain with families

Becoming a burden for family members was voiced as an important issue, in particular in Hong Kong. As illustrated in the following quotes, it even became existential.

I am unhappy because I have done less housework … I once thought that it could be better if I died. Don’t worry, I won't commit suicide. However, I think in this way, maybe it would be better if I die. I won't commit suicide because I don't have the courage to do it … I feel like I am giving them trouble. For example, when I tell them (family members) that I have LBP, they will just reply “Be careful and do more exercise!” They don't understand that it's useless to do more exercise … I am afraid of becoming a burden to them. If I can't walk anymore, they will need to assist me to go to the toilet. Many things will be affected, including my daily life. (HK06_F_75)

My children seldom visit me because they are busy. They only make phone calls with me during holidays. They only come back to have dinner when it is my husband's birthday. They rarely visit us … I rarely chat with my family members. I don't want them to worry about me. Sometimes my daughter will find someone to take care of us [interviewee and her husband]. However, my home has little space. Hence, I don't want her to find someone … I seldom discuss my things with others. I don't want to increase their burden. Further, there's nothing they can do to help me. I rarely talk about this with my children. It's not necessary to talk about this. (HK04_F_81)

Very interestingly, sometimes I feel helpless due to the intense pain. (HK03_F_75)

Family structure and respect for elders are inherent to the culture in Hong Kong. The importance of family is a core social value for Chinese, and is consistent with collectivism within this culture. Sung (41) argues that filial piety (responsibility, respect, sacrifice and family harmony) influences children's attitudes towards their elders. While one participant (HK06_F_75) saw herself as a burden, another participant (HK04_F_81) deplored that her children did not have time for her.

### Social roles

Results revealed the importance of social roles for the older adults with chronic LBP. This theme had different aspects. While our participants indicated that they aimed to maintain their roles within their families, they also insisted on the fact that their families supported them, both psychologically or financially. In particular in the Hong Kong context, social roles were clearly described with children supporting their parents.

#### Maintaining their roles in families (e.g., housework)

It was considered a role of a spouse to take care of the partner and the children, as illustrated in the quotes from a Hong Kong and a Swiss culture.

Now I need to take care of my old husband. I often use the wheelchair to bring him to the day care centre or wait for public transport. Sometimes when I bring him back with wheelchair, I will hold my lower back. (HK04_F_81)

Well, there were times when I said to myself “Well, you've lived your whole life with pain”, and I'm quite proud of myself for not having poisoned the life of my husband or my children, because I don't complain about it. (…) They didn't believe me when I was a child. So why am I still sharing the live with those I love. I have never made a misery. I put up with it, I've always put up with it. (S12_F_74)

Expectations about social roles were put forward in both regions. As mentioned in the introduction, Leventhal et al. (33) argue that representation of health and illness are shaped by the socio-cultural background. With regard to women's role in society, taking care of family members might be considered as a ubiquitous social role, independent of the cultural background.

#### Experiencing supports from family and friends

Family members and friends play an important role, either by helping in day-to-day activities, giving advice, or supporting the older adult financially.

At that time my husband was still alive. He bought the cooking ingredients and cooked meals for us. I was in severe pain … My sons also give me money from time to time … My son told me I can live longer if I am careful. The most important thing is to stay healthy. (HK11_F_81)

Three sons sent me to a hospital. I was sent to the hospital very early. Here is close to the Hong Kong Adventist Hospital in Tsuen Wan. Therefore, I received treatment there. My children brought me there. (H08_F_80)

They just reminded me to be careful when I am eating and walking. (HK06_F_75)

My friend also take care of me from time to time. My friend cooks meals for me. (HK12_F_84)

My wife is here, if I have to put some of the cream on my back, well, she can do it. (S06_M_73)

A Swiss participant mentioned that some of his close friends actually did not know about his chronic pain.

Many do not know that I have low back pain, many do not know. That is- I went this morning to a gathering [Apéro] at the lake with friends. Then I said that I have to go as I have an appointment with the physio this afternoon and an interview with question mark [pointing to interviewer, laughs]. “Ah what do you have with the back? Why do you need to go to the physio? Then I said: “another time” because I really had to go. But many do not know, as well people who are quite close to me, they do not know. (S05_M_72)

Maintaining social roles within family was considered important by participants. However, depending on the cultural context, some information regarding their health problem was not shared widely within the outer circle of family.

#### Being content despite LBP - coping

Participants, even though in pain and with limitation of activities, still felt rather optimistic and content. In addition, it was important for them to receive effective treatment for their back pain.

I am an optimistic person. I have been optimistic since I was young. I don't feel sad … I won't be unhappy. I am satisfied. I have food and shelter. I won't ask for more … I am not worried about it. (HK07_F_85)

I'm not worried. The most important thing is to receive effective treatment. It's great to receive treatment in Yan Chai Hospital, because it's not too expensive. (HK09_F_76)

I've always had a good life, despite the pain. So it hasn't affected my morale. If it affects me, if it disturbs my morale, it's when I have other concerns, but not for my back. The back, I came into the world with it, so I'm going to leave with it. (S12_F_74)

The body is part of the man, the human being. (…) Without body there is no psyche, it is part of it. The two-, it's the two elements, as we say the body and the soul. So, the body is what accompanies the soul, it's the two. I believe in the anima, in the soul, that means that when there are psychological problems, you perform physical activity, and you forget. And when you have physical pain, you try to escape through intellectualism, and then like that you find a balance. (S07_M_73)

Participants chose an active approach to improve the situation: as one participant considered herself a “hardworking individual” – as illustrated in the next quote – she will do her exercises (HK03_F_73) because she wanted to avoid surgery. Another participant would go for a walk even when tired (HK07_F_85).

I think it was more related to my physical condition. I believe that I need to accept the fact and find methods to improve the situation. I am hardworking because I don't want to do the back surgery. I know I won't be able to do exercise after I return home today, that's why I already did the exercises before lunch. (HK03_F_73)

I only feel tired. It's not a severe disease. It's fine if I take some rest. Nowadays I don't work anymore. When I feel tired I will go to sleep. When I wake up, I will take a walk if I am still tired. The back fatigue doesn't matter.” (HK07_F_85)

It was also mentioned that the Swiss health insurance provided the services they needed to get better.

Well, listen, except that… When we are insured, we are very well insured. But that's since the moment when we retire. Except for our doctor, we're very happy. We don't need anything else. We have our own medication. (S12_F_74)

The theme presented here reflects how participants adapted to the fact that they experienced LBP but they also gave meaning to other aspects of life or rely on quality healthcare services in order to get better.

#### Enjoying social activities

As most of the Hong Kong participants were interviewed at an elderly centre, some of them expressed their opinion on social activities, which tended to be positive.

There were many “kaifongs” (街坊 ; neighbours). I went to Cantonese restaurant with them. (HK01_F_83)

Now I am just a volunteer. For example, I am a helper in a dancing class. (HK08_F_80)

In Switzerland, social activities were also considered important, as some of the participants enjoyed traveling (S03_M_75) while others went hiking and ski touring.

Well I go as well- we go out, we have the general ticket [*allowing for free train travel all around Switzerland*] and then we take often the train. The pain – it is little actually during the day, that I am limited. (S03_M_75)

It depends on the time and the form of the crisis. Yes, that's it. And when I walk, I really like walking. But if I go for more than two and a half, three hours, then it really starts to pinch and pull down my leg. That's it. […] And in the end, I really enjoy walking and admiring the landscape. […]We walk in the snow, where the snow carries us. (S12_F_74)

For example, I don't do downhill skiing anymore because it gives a lot of jolts in the lower back, among other things, and that doesn't suit me. I do ski touring still quite regularly when it's good, when there's snow. And since I belong to the Alpine Club. (S13_M_78)

Social activities helped older adults with chronic LBP to continue being part of a group, be it by volunteering (HK08_F_80), going to a restaurant (HK01_F_83), traveling (S03_M_75) or enjoying activities in nature (S12_F_74 and S13_M_78).

## Discussion

The aim of this collaborative research project between Switzerland and Hong Kong was to illuminate older adults' experience of living with chronic LBP and to describe the implication of chronic pain on older adults' daily life in Western and Eastern cultures. The findings revealed a complex interaction between individual and social consequences of chronic LBP in an aging population. The following three subsections will summarise the study results: (1) adjustments and adaptations for activities of daily living; (2) social support and cultural influences; and (3) differences of healthcare systems in Hong Kong and Switzerland.

### Adjustments and adaptations needed for activities of daily living when living with chronic LBP

While chronic LBP is known to adversely affect the well-being of patients, the types of responses and/or severity of distress/suffering experienced by different individuals vary considerably. Given that chronic pain is not purely a physical problem (42), the multidimensional interactions among biological, psychological, and social factors often result in differential experiences of individuals. Since older adults need to face various age-related physical and psychosocial changes (e.g., decreases in pain sensitivity or fitness (14, 43, 44), altered life goals and social roles, or reduced/terminated monthly income), these changes may affect the ability or experiences of older people in managing chronic LBP (45, 46). Similarly, the presence of chronic LBP in older adults may lead to fear avoidance behaviour, concerns about underlying severe pathology (47), reduced physical activity and deconditioning, or compromised social and family functioning (48, 49). Therefore, clinicians not only need to use age-specific assessment tools to comprehensively evaluate the condition of older people with LBP (50), but also need to educate these patients and/or their caregivers regarding the aetiology and trajectory of chronic LBP, as well as potential coping strategies.

Our study revealed that older adults with chronic LBP coped with their pain differently. While some people were pessimistic about their condition, others dealt with their pain positively. Although their differences may be attributed to individual personal traits, it may also be ascribed to their differential acceptance of living with chronic LBP (like other chronic diseases). Leventhal et al. (33) argue that illness representation and coping strategies are important to understand how people adapt to their physical problems. As socio-cultural background influences the way people with chronic pain live and manage their pain, it is important to uncover the underlying cultural beliefs and values. In order to facilitate older adults with chronic LBP to effectively cope with their pain and optimize their physical function, clinicians should educate these patients regarding the characteristics of chronic LBP (e.g., central pain amplification, psychological influences, and pain without biological value), provide proper self-management techniques, and manage their expectations (49, 51). For example, exercise is an evidence-based intervention for patients with chronic LBP (52–54) recommended by multiple clinical guidelines (55, 56). It is essential for older adults with chronic LBP to understand this important active approach in managing their pain. Other techniques, such as pacing and goal setting are useful strategies for the self-management of chronic pain (57). Given that chronic LBP is always associated with psychosocial issues, new cognitive behavioural therapy approach, namely acceptance and commitment therapy has been shown to be effective in improving clinical outcomes of older adults with chronic pain (58–61). This novel approach uses metaphors, experiential exercises, mindfulness practice, and cognitive defusion to help older adults accept the good and difficult feelings/emotion associated with pain in the journey of pursuing values-based action (e.g., a fulfilling family role). Through multimodal approaches, older adults with chronic LBP can better manage their pain and associated psychological health.

### Social support and cultural influences

Results revealed that maintaining social roles is important, which aligns with the findings from a systematic review of qualitative studies exploring patients' experiences of chronic LBP (62). Although the patients included in the review were not exclusively older adults (the age range was 17 to 84), the analysis revealed that relationships with family and friends was a key theme identified across all 27 studies. Specifically, friends and family provided emotional and tangible support, and helped to moderate “negative lower mood or even depression” (p. 292). At the same time, maintaining their social roles in the presence of chronic LBP related disruptions was identified as a problem and challenge that resulted in negative emotions such as guilt (e.g., feeling like a burden) and anger. The researchers also found that only one study highlighted the need to understand chronic LBP within the context of culture (e.g., cultural values in Iran that emphasize “duty to family” that contributed to the stress of the patients in juggling their LBP with their respective roles in the family).

The findings of the current study suggest that there is a need to take into consideration differences related to cultural aspects and society in order to help older adults with chronic LBP. In the Hong Kong context, co-residency of multiple generations is a common practice due to traditional cultural Confucian values (63) and financial reasons (e.g., Hong Kong has one of the least affordable housing markets in the world) (64). While this setting may provide good family supports, it may cause a lot of conflicts or confrontations if the needs of older adults are not met or the different generations have conflicting views on family roles and obligations (64, 65). For example, Holroyd (2003) found in her ethnographic study examining caregivers' perspectives toward chronically ill older adult members in Beijing and Hong Kong, that in both contexts, there was an emerging trend transitioning away from “Confucian-informed duty-centered family obligations to one in which obligations are now centered on relationships” (65), p. 316).

In Switzerland, the generation of the 65 + years old is still quite traditional, yet autonomous when it comes to their life choices. In particular, women maintain their tradition to take on the role of “guardian of family and extra-family relations” (66). Yet, the society is changing, with only 1% of households constituted with more than two generations, and 32% of older adults living alone (66). In the Swiss culture where self-determination is important, friends and a wider social circle can sometimes replace family relations. Therefore, differences regarding what is considered an acceptable social circle to discuss health issues (close family, friends, community) should be taken into consideration when designing interventions for older adults with chronic LBP.

Collectively, culture can play an influential role in shaping the experience of chronic LBP among older adults. Future research should examine the role of culture in shaping the chronic LBP experience, not only cross-culturally but also between generations.

### Comparison between Swiss and Hong Kong healthcare systems

As mentioned above, differences between Switzerland and Hong Kong were related to social support networks but also to opportunities/offers from the health and social system, thereby impacting individual experiences with chronic LBP. As discussed in the introduction, the Swiss and Hong Kong healthcare systems are both of high quality; yet, access to services can become problematic in Hong Kong, but not in Switzerland.

More specifically, three quarter of the Swiss population between 65 and 74 years perceives their general health as good or very good, the best score in comparison with other European countries (67). Höpflinger argues that the health status of older people is closely associated with economic and socio-political factors and that the social and economic safety is related to the health of the aging population (67). The Swiss health care system (even though very expensive) allows for an excellent coverage of healthcare needs of the population.

Looking at the social security and health insurance schemes in both regions, it can be noted that there are remarkable differences. While Hong Kong's public healthcare system is quite efficient and cheap, yet with long waiting times, access to the Swiss healthcare system is unproblematic but can be very costly (e.g., out-of-pocket payment, co-payment, deductible, etc.). In particular, adults aged 80 years or older are in need of supplementary payments from the Old Age Insurance because they do not have sufficient funds to cover their healthcare costs (66). Financial hardship was part of the discourse in our study.

In sum, until now, multiple studies identifying factors that are related to or may modify chronic LBP outcomes in older adults have some limitations as they often used self-reported questionnaires to examine the influences of chronic LBP in older adults (68), without listening to the concerns or needs of older adults with chronic LBP (69). Although some qualitative studies have been conducted to investigate lived experiences of patients with chronic LBP (70–73), most of them did not focus on the experiences or needs of older adults. This qualitative study was able to shed light on consequences of chronic LBP for older adults allowing for a better understanding of their needs, and taking into consideration the contexts in which they live and receive health care services. While some qualitative studies have investigated the impacts of chronic LBP on various facets of life (e.g. coping strategies, social roles, attitudes towards treatments) in older adults (74, 75), often these studies investigated health issues in older adults residing in a specific setting, such as Nursing homes (68, 76) but not simultaneously in two different cultures. This qualitative research examined the distinct challenges, concerns or experiences faced by older adults with chronic LBP living alone or with family in the community setting, aiming to identify cross-cultural specificities in order to be able to better plan and implement adequate health and social service interventions. Given the growing trend of globalization and global mobility, it is not uncommon for people from different cultures to live in the same region or country. Clinicians should be aware of cultural differences (e.g., self-perceived roles in a family) in older adults, and provide proper medical services and psychosocial supports to older people with chronic LBP so as to enhance their abilities in self-managing chronic LBP.

The current results are crucial as they are the first step for developing and evaluating complex interventions according to the Medical Research Council's framework (77). Developing effective and culturally sensitive interventions for an older adult population experiencing chronic LBP can be challenging and, therefore, a systematic approach is required to propose an effective, feasible and person-centred intervention. The systematic methodological approach allows for the identification of important aspects crucial for successful interventions for older adults with chronic LBP.

Some limitations of this study should be noted. Participants enrolled in this study were selected through different gatekeepers: In Switzerland, medical and physiotherapy ambulatory practitioners were referring participants, while in Hong Kong community centres were the site of recruitment. These two different approaches are not comparable and might make comparison across the two regions more challenging. However, results indicate that there were common themes across regions despite different socio-economic status and professional background. A second challenge was related to the fact that the research team worked across languages and long distances, in particular, the pandemic did not allow for research teams to engage in face-to-face interactions as planned and had to resort to video-conferences, shared NVivo analyses and online documentation.

An interesting and noteworthy point of discussion from this cross-language study is related to translation. Our research team had several discussions regarding the role and implications of translation in our study, and in particular what paradigm we should adopt. On the one hand, for example, there is a common view in the literature that the translation process is technical and neutral, where the task simply involves exchanging words from one language to another, verbatim (78). On the other hand, there is the view that in cross-language research, the strive towards equivalence may not be ideal (79). There are words, phrases, socio-cultural idioms, and proverbs that exist in different languages that cannot be translated. Further, scholars like Temple (79) warned against “the linguistic imperialism central to the unquestioning use of English as a baseline language” (p. 847). Our team had numerous discussions and reflected on our own understandings and use of multiple languages. For example, it is noted here that our team is very unique in terms of (inter-)cultural competencies because one researcher is a Canadian Chinese, while another researcher is a Hong Kong-born Chinese who lived in North America for approximately six years. Therefore, the Hong Kong researchers are bilingual researchers who can communicate fluently in English. Meanwhile, on the Swiss team, one researcher speaks German, French and English, as well as has lived in the USA and Hong Kong for approximately seven years each. The other Swiss researcher is also trilingual (French, German, English) having lived in several European countries, therefore allowing for an in-depth understanding of languages and cultures. Our unique multi-cultural research team enabled the discussion of codes and concepts with ease.

The implication for other qualitative researchers who are interested in conducting cross-cultural studies is that they should collaborate with the right multi-lingual and (inter-cultural) collaborators in the countries of interest (especially for low-income countries where the English proficiency of local researchers may be relatively low). During our discussions, we came to the agreement that the paradigm suggested by Temple (79) should be adopted for our study. Overall, this experience prompted us to deeply reflect on the role and impacts of translation in future cross-language studies, and on strategies to enhance the translation process and to mitigate negative effects of it.

## Conclusion

Although chronic LBP may negatively impact older adults in different aspects, individual approaches as well as social and health system supports influence older adults' attitude toward their pain and self-management strategies. Themes identified were related to negative perceptions/experiences: (1) interferences of daily function; (2) pessimistic attitudes toward their conditions/prognosis; and (3) self-perceived burden related to families. Conversely, four themes revealed attributes to social roles: (1) maintaining their roles in families; (2) experiencing supports from family and friends; (3) being content despite LBP; and (4) enjoying social activities. Developing effective and culturally sensitive interventions for an elderly population with chronic LBP can be challenging but is essential for the development of innovative healthcare services tailored to the population's needs. The methodological approach used for this research project establishes a framework for developing and evaluating complex interventions.

## Data Availability

The raw data supporting the conclusions of this article will be made available by the authors, without undue reservation.

## Appendix

**Interview Guide**

**Pain experience**

1. When you think about the last time you had back pain, can you tell me how it feels like?
  **Impact on daily life**
2. Can you tell me about your experience of back pain and how it affects your life?
3. What are the experiences in living with chronic back pain?
  *For Yourself*
  *For Your friends and family*
  *For Your relationships with your healthcare providers (physician, nurse, therapists, …)*
4. What bothers you the most about your back pain?
  **Representation of health and illness**
5. What do you think might cause your back pain?
6. What is in your opinion the evolution of your back pain?
  **Coping strategies**
7. How do you manage your back pain?
  **Closing question**
  Is there anything you would like to tell us that we have not covered yet in this interview?
